# Supplementary material for: The interactome of intact mitochondria by cross-linking mass spectrometry provides evidence for coexisting respiratory supercomplexes
Source: Mol Cell Proteomics. 2017 Dec 8;17(2):216–32. doi: 10.1074/mcp.RA117.000470 (PMC5795388; doi:10.1074/mcp.RA117.000470)
Supplement: Supplemental Data [file supp_RA117.000470_133922_0_supp_23978_fzffwf.zip › spectra_annotation/mito_spectra_annotation/153-1-4-1-5-1.pdf]

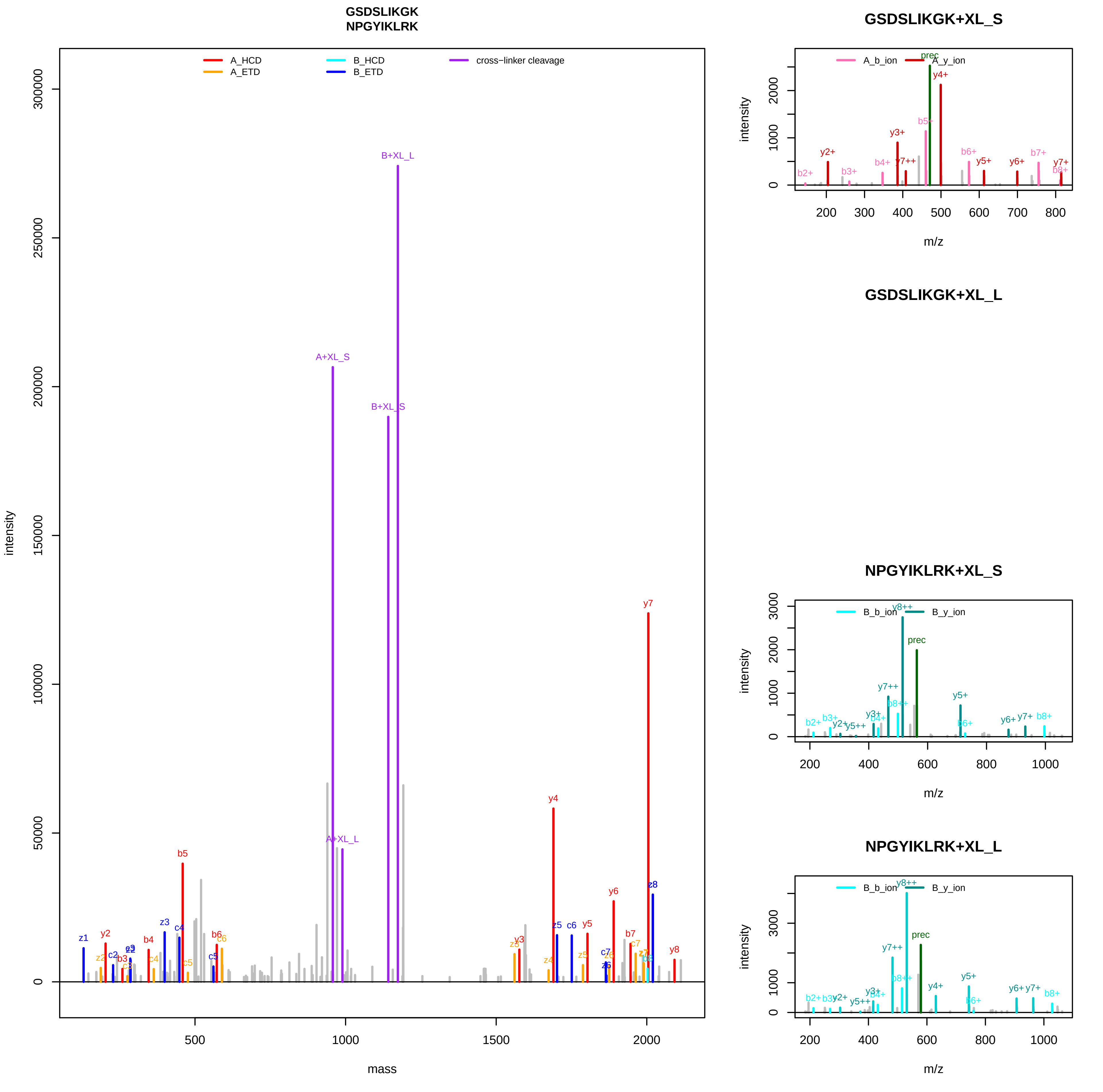
GSDSLIKGK
NPGYIKLRK
A_HCD
A_ETD
B_HCD
B_ETD
cross−linker cleavage
intensity
mass
B+XL_L
A+XL_S
B+XL_S
A+XL_L
y7
y4
b5
y6
z8
y2
z1
z3
c4
b6
c6
y5
z5
c6
b7
c7
y8
y3
GSDSLIKGK+XL_S
A_b_ion
A_y_ion
prec
y4+
y3+
b5+
y2+
b3+
b2+
b4+
y7++
b6+
y5+
y6+
b7+
y7+
b8+
m/z
GSDSLIKGK+XL_L
NPGYIKLRK+XL_S
B_b_ion
B_y_ion
y8++
y7++
b8++
y5+
y3+
b4+
b2+
b3+
y2+
y5++
b6+
y6+
y7+
b8+
NPGYIKLRK+XL_L
y4+
